# Synthesis of Helical Carbon Fibers and Related Materials: A Review on the Past and Recent Developments

**DOI:** 10.3390/nano5020937

**Published:** 2015-06-02

**Authors:** Himanshu Raghubanshi, Ezekiel Dixon Dikio

**Affiliations:** Applied Chemistry and Nanoscience Laboratory, Department of Chemistry, Vaal University of Technology, P. O. Box X021, Vanderbijlpark 1900, Republic of South Africa; E-Mail: hraghubanshi@rediffmail.com

**Keywords:** synthesis, catalyst particle, helical carbon fibers (HCFs), growth mechanism, composite material

## Abstract

Helical carbon fibers (HCFs) have been widely studied due to their unique helical morphology and superior properties, which make them efficient materials for several potential applications. This review summarizes the past and current advancement on the synthesis of HCFs. The review focuses and discusses synthesis strategies and effect of experimental parameters on the growth of HCFs. The effect of preparation method of catalyst, catalyst nature, catalyst composition, catalyst size, catalyst initial and final shape, reaction temperature, reaction time, carbon source, impurities, and electromagnetic field on the growth of HCFs is reviewed. We also discuss the growth mechanism for HCFs and the synthesis of HCFs related materials. Finally, we conclude with a brief summary and an outlook on the challenges and future prospects of HCFs.

## 1. Introduction

**Figure 1:**
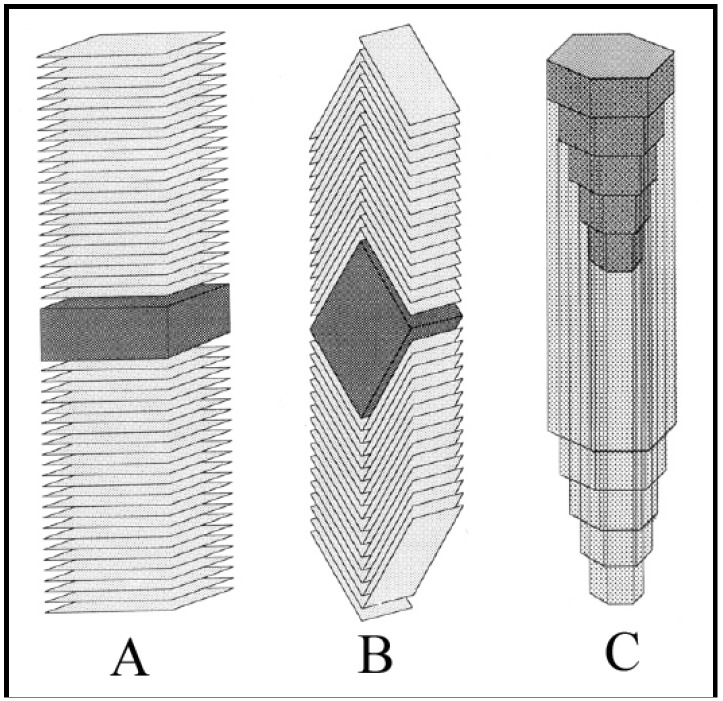
Schematic representations of carbon nanofibers (CNFs): (**A**) “platelet”; (**B**) “herringbone”; and (**C**) “tubular” ([6], Rodriguez *et al*., 1995).

**Figure 2:**
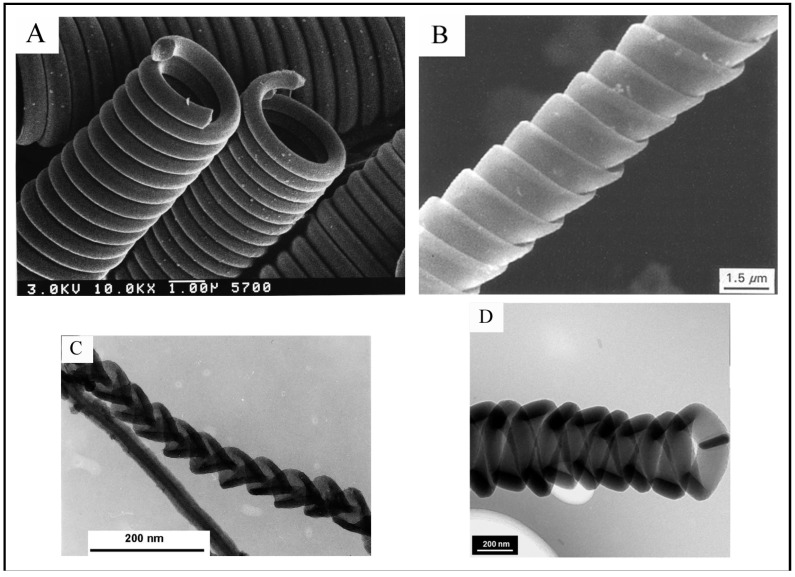
Various morphologies of carbon fibers: (**A**) double-helix regular circular carbon coils ([7], Motojima and Chen, 1999); (**B**) regularly ribbon-like flat coiled carbon fibers ([13], Motojima *et al*., 1995); (**C**) nanobraids (together with a carbon nanofiber) ([14], Liu *et al*., 2003); and (**D**) intertwined carbon fibers with symmetrical growth mode and centrally located Cu catalyst particle ([15], Shaikjee *et al*., 2011).

Carbon is a very interesting element owing to its ability to form different structural forms (allotropes). The valency of carbon is a major characteristic feature, which enables it to form various allotropes. Diamond and graphite are the well-known allotrope of carbon. In the last few decades, a large number of allotropes and variants of carbon have been discovered; some of them are carbon filaments/fibers (discovered in 1889) [1], fullerenes (discovered in 1985) [2], carbon nanotubes (CNTs) (discovered in 1991) [3], graphene (discovered in 2004) [4], *etc*. Carbon can be tailored into the above said allotropes, particularly those in the nanometer range, by changing the synthesis methodology. Graphene sheet is the basic building block for these carbon nanovariants. In 1889, Hughes and Chambers reported a method for the growth of carbon filaments for the first time [1]. Thereafter, extensive analysis has been carried out by Baker *et al*. in 1972 for the growth of carbon filaments [5]. These carbon filaments are a kind of solid-cored fiber with cylindrical structures; named as carbon fibers (CFs). Depending on the diameter, CFs can be categorized into carbon nanofibers (CNFs) and carbon microfibers (CMFs). CNFs possess the following microstructural configuration, *i.e*., (i) platelet CNFs, wherein the graphite sheets are perpendicular to the fiber axis; (ii) herringbone CNFs, wherein the graphite sheets are inclined at an angle with respect to the fiber axis; and (iii) tubular CNFs, also named CNTs, wherein the graphite sheets are parallel to the fiber axis (Figure 1) [6]. From their morphological point of view, CFs can take various shapes such as planar, branched, twisted, spiral, coiled, helical, *etc*., some of them are shown in Figure 2.

**Figure 3:**
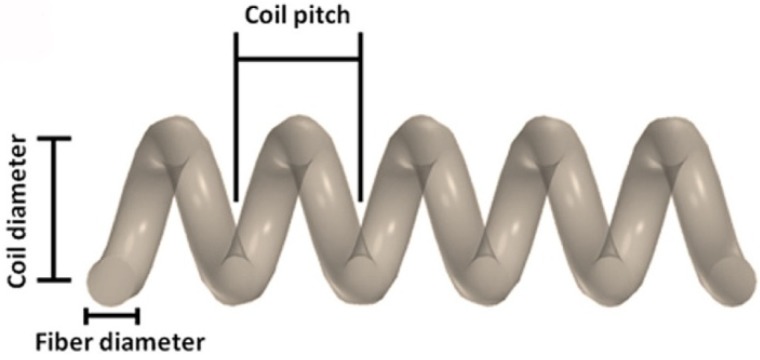
Schematic illustration for fiber diameter, coil diameter, and coil pitch ([16], Shaikjee and Coville, 2012).

Helical carbon fibers (HCFs) are very fascinating new carbon material, composed of fibers with “helical” morphology. Their three-dimensional helical/spiral structure is a fundamental structure in nature like great maelstrom of the cosmos, α-helix of proteins, double helix of deoxynucleic acid (DNA), screw dislocation in solids, electric waves, growth of vine plants, screw dislocation in solids, *etc*. [7]. Helical morphologies of CFs has been named double/single-helix CF, micro/nano coiled CF, circular/flat coiled CF, spiral CF, helical CF, twisted CF, *etc*., by various researchers depending on their morphologies. In this review, we will use the notation HCFs for these various types of helical morphologies of carbon. Kuzuya *et al*. categorized HCFs into helical CNFs (HCNFs) and helical CMFs (HCMFs), according to the coil diameter [8]. Typical fiber diameter, coil diameters and coil length in HCNFs are 50‒200 nm, 50‒1000 nm, and 0.3‒3 µm, respectively; and in HCMFs are 0.3‒1 µm, 1‒10 µm, and 1‒10 mm, respectively [8,9,10,11,12]. Schematic illustration for fiber diameter, coil diameter, and coil pitch are shown in Figure 3.

HCFs have attracted much interest due to their interesting helical structures, unique properties, and potential applications [17,18,19,20,21,22]. Their properties include high: chemical stability, surface area, elasticity [17,21,23], mechanical strength [24], and good thermal and electrical conductivity [24,25,26]. HCFs have shown excellent electron field emission characteristics [27,28,29,30] and wide band absorption of electromagnetic waves [31,32,33,34,35]. Because of their unique morphology and properties, HCFs have been proposed for potential applications in polymer additives [36,37,38], supercapacitor electrodes [39,40], catalysts [41], catalyst supports [42,43], hydrogen adsorption [39,44,45], generator or detector of magnetic field [46], an electrical inductors [21], micro/nanospring [47], sensors [21,48,49], nanodevices [50], *etc*.

Twisted thin CFs in the form of a rope has been reported by Davis *et al.* in 1953 [51]. Also, the growth of vapor phase HCFs was reported by some other researchers [52,53]. Thereafter, the detailed and pioneering works on the synthesis of HCFs were first introduced by Motojima *et al*. [8,12,17]. Furthermore, many groups have employed chemical vapor deposition (CVD) to grow HCFs via catalytic decomposition of hydrocarbons, employing many different metal catalysts [54,55,56,57,58,59]. Till date, extensive research has been done on the synthesis [8,50,60] and mass production [50,61] of HCFs. The catalysts were usually selected from transition metal particles such as Ni [17,59,62], Fe [9,57,63], Co [64,65], Cu [38,66,67,68] and also from their mixture [69] as well as alloys [15,70,71,72,73]. These transition metal nanoparticles act as nucleation centers for the growth of CFs and catalyze the decomposition of the carbon source [5]. The catalytic activity or capability of carbon deposition and condensation, however, is not the same for all metals.

During the last decade, HCFs-based materials such as metal particles decorated HCFs, and composites of HCFs have also been extensively synthesized and studied for their potential applications in the fields of electrocatalyst [74,75], catalyst for hydrogen storage materials [72,76], sensors [77], electromagnetic wave absorbers [78], *etc*. The synthesis, properties and possible potential applications of HCFs have been reviewed by some researchers [16,79,80]. The aim of this review article is to elaborate the brief history and recent advancement on the synthesis of HCFs and related materials. We briefly cover the synthesis methodology and the effect of experimental parameters on the growth of HCFs.

## 2. Synthesis of Helical Carbon Fibers (HCFs)

A variety of methods have been explored for the synthesis of planar CFs such as arc discharge, laser vaporization and CVD. Among various preparation processes, CVD has been found to be the most efficient method for the synthesis of HCFs [60,81,82]. Mostly planar CFs have been obtained as a final product while using arc discharge and laser vaporization methods for the synthesis of carbon materials owing to their high synthesis temperature (>2000 °C) [83]. Vaporization of solid graphite for CFs growth require high growth temperature since carbon atoms gets higher mobility and results nearly perfect hexagonal graphite for the growth of planar CFs [83]. Conversely, carbon atoms get lower mobility in CVD method since, generally, the synthesis temperature is in the range of 400‒1000 °C. The lower mobility of carbon atoms favors the formation of non-hexagonal graphite in the growing nucleus; and results a poor crystalline graphitic structure and formation of HCFs [83]. In addition to this, CVD provides easy control on the experimental parameters, which control the CFs growth [84,85]. However, except CVD, HCFs have also been synthesized using arc discharge [86], flame synthesis [87], and hydrothermal synthesis [88]. It should be emphasized here that the arc discharge, laser vaporization, and electrospinning, which are usually used in the synthesis of planar CFs, are generally not suitable for the preparation of HCFs. Since, reproducibility and yield of HCFs in these methods is low compared to CVD process and also these methods are costly compared to CVD. Table 1 shows the growth conditions for the synthesis of HCFs used by various researchers.

**Table 1:** Growth conditions for the synthesis of helical carbon fibers (HCFs).

S.No.	Carbon Source	Carrier gas		Promoter	Catalyst	Substrate	Temperature	Method & Final Product	Coil diameter & Length	Ref.
1.	C_2_H_2_	N_2_		H_2_S	Thin films of Au metal or Au/Ni alloy	Graphite plate	700‒740 °C	CTCVD; CNCs	50‒450 nm; 0.3‒3 µm	[8]
2.	Acetone dissolved C_2_H_2_	H_2_, Ar		Thiophene, PCl_3_	Ni powder	Graphite	600‒800 °C	CTCVD; CCFs	3‒5.4 µm; µm order	[9]
3.	Acetone dissolved C_2_H_2_	H_2_, N_2_		Thiophene	Ni powder	Graphite	750 °C	CTCVD; CMCs	µm order; µm order	[10]
4.	C_2_H_2_	H_2_, N_2_		H_2_S	Fe alloy (54Fe-38Cr-4Mn-4Mo)	Graphite	750‒790 °C	CTCVD; Spring-like CNCs	100‒1000 nm; Several hundred µm to 1 mm	[12]
5.	C_2_H_2_	Ar		---	Ni plate & powder	---	350‒750 °C	CTCVD; Coiled carbon filaments	2‒8 µm; 0.1‒5 mm	[17]
6.	C_2_H_2_	Ar		---	Cupric tartrate	---	380 °C	CTCVD; HCFs	0.5‒1 µm; µm order	[38]
7.	C_2_H_2_	H_2_		---	Ni nanoparticles	Ceramic plate	450 °C	CTCVD; CNCs	120‒500 nm; 30 µm	[50]
8.	C_2_H_2_	H_2_, Ar		---	Au and K metals	Graphite foils	450 °C	CTCVD; CNCs	110‒140 nm; 10 µm	[54]
9.	C_2_H_2_	H_2_, N_2_		Thiophene	Ni nanoparticles	Graphite plate	750 °C	CTCVD; single-helix carbon nano/micro coils	150 nm‒3 µm; µm order	[55]
10.	C_2_H_2_	---		---	Nano Cu	---	250‒400 °C	CTCVD; HCNFs	100 nm‒10 µm; µm order	[61]
11.	C_2_H_2_	H_2_, N_2_		Thiophene	Ni nanoparticles	Graphite	750 °C	CTCVD; CCFs	3 µm; ---	[62]
12.	C_2_H_2_	N_2_		---	Co	Silica gel	720 °C	CTCVD; Coiled CNTs	nm order; µm order	[65]
13.	C_2_H_2_	H_2_, Ar		---	Cu nanoparticles	---	350 °C	CTCVD; HCNFs	80 nm; µm order	[67]
14.	C_2_H_2_	---		---	Cu nanoparticles	Si wafers	260 °C	CTCVD; CNCs	80‒120 nm; µm order	[68]
15.	C_2_H_2_	H_2_, He		---	Ni nanoparticles	---	650 °C	CTCVD; HCNFs	~150 nm; 6‒8 µm	[72]
16.	CH_4_	Ar		---	Co+Cu alloy film	SiO_2_/Si	600 °C	Hot filament CVD; Carbon helix nanofibers	20‒500 nm; µm order	[73]
17.	C_2_H_2_		He	---	Fe films	Indium tin oxide-coated glasses	700 °C	CTCVD; CNCs	nm order; µm order	[81]
18.	C_2_H_2_		Ar	Ammonia	Na_2_CO_3_	Ceramic boat	500 °C	CTCVD; *N*-doped CNCs	---	[82]
19.	C_2_H_2_		---	Sulfur and phosphorus	Ni	Metal Plate	At various gas pressures	Arc-discharge; Carbon coils	6‒10 µm; 50‒80 µm	[86]
20.	Acetone dissolved C_2_H_2_		H_2_, N_2_	Thiophene	Fine powder of WS_2_	Graphite/ stainless steel plate	775‒785 °C	CTCVD; CMCs	microns; millimeters	[89]
21.	C_2_H_2_		H_2_, Ar	---	Ni	SiC	700 °C	Microwave CVD; Micro CCFs	---	[90]
22.	C_2_H_2_		Ar	---	NaNO_3_	Ceramic plate	450 °C	CTCVD; HCNFs	nm order; µm order	[91]
23.	C_2_H_2_		---	---	Cu nanoparticles	---	195 °C	CTCVD; HCNFs	100 nm; 1 µm	[92]
24.	C_2_H_2_		H_2_, Ar	---	Pd nanoparticles	C_60_	550 °C	CTCVD; HCNFs	nm order; < 10 µm	[93]
25.	C_2_H_2_		---	---	Cu	MgO	237 °C	CTCVD; HCNFs	100 nm; µm order	[94]
26.	C_2_H_2_		H_2_	Thiophene	Ni foam	Ni foam	973‒1073 K	CTCVD; CMCs	1.5‒2.5 µm; ---	[95]
27.	C_2_H_2_		H_2_, N_2_	Thiophene	Ni	Activated CNTs	650‒800 °C	CTCVD; Carbon micro and nano coils	4-7 µm, ---; 200 nm, ---	[96]
28.	C_2_H_2_		N_2_		Ni-sulfide	---	740 °C	CTCVD; CMCs	3‒4 µm; millimeters	[97]
29.	C_2_H_2_		N_2_	Thiophene	Ni powder	---	1100 °C	CTCVD; Double helical carbon micro coiled fibers	3‒4 µm; millimeters	[98]
30.	C_2_H_2_		---		Cu	---	240 °C	CTCVD; HCNFs	nm order; ---	[99]
31.	C_2_H_2_		---		Fe nanoparticles	---	475 °C	CTCVD; Helical CNTs	120‒200 nm; µm order	[100]

### 2.1. Chemical Vapor Deposition (CVD)

CVD is a chemical process in which catalyst and or substrate is exposed to one or more volatile precursors. By reactions or decomposition of precursors on catalyst/substrate surface, high quality, high-performance, solid materials can be produced. Till now, several CVD-based methods have been developed to produce high performance materials.

#### 2.1.1. Catalytic Thermal CVD (CTCVD)

**Figure 4:**
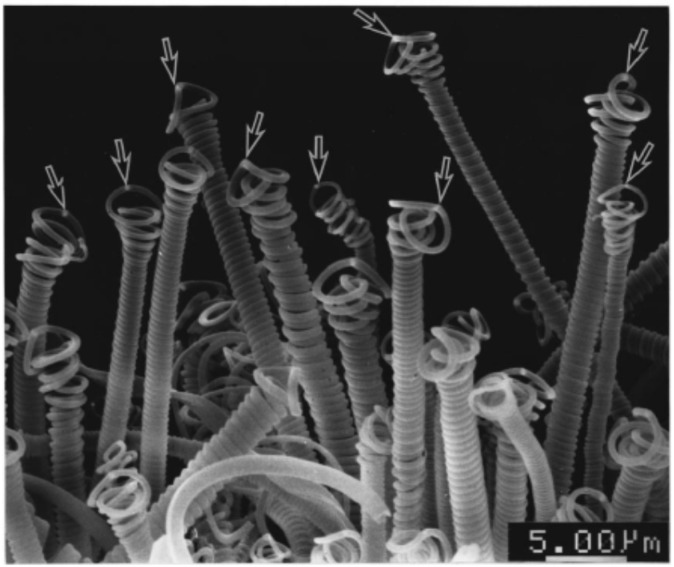
Representative carbon coils growing vertically on the substrate ([7], Motojima and Chen, 1999).

Catalytic thermal CVD (CTCVD) process involves the pyrolysis of a hydrocarbon (e.g., methane, acetylene, ethylene, *etc*.) over transition metals (e.g., Fe, Co, Ni, *etc*.) as catalysts at high temperatures (500‒1000 °C) to produce carbon nanomaterials. Most of the researchers have been used CTCVD process for the growth and finding the optimal condition for HCFs on a large scale. CTCVD method was first used by Motojima *et al*. in the early 1990 to synthesize regularly coiled carbon filaments by Ni-catalyzed pyrolysis of acetylene containing a small amount of thiophene as an impurity at temperature of 350‒750 °C [7,17]. In this study, Motojima *et al*. found that these regularly CFs consist of pair-coiled fibers, and were elastically extended up to about three times of the original coil length [17]. The carbon coils were grown perpendicularly, pointing in the direction of the source gas inlet, on the graphite substrate as shown in Figure 4 [7]. Here, a fine Ni grain material was used as catalyst and always observed on the tip of all the carbon coils (arrows in Figure 4). The regular carbon coils have a coil diameter of 3‒6 µm, coil pitch of 0.5‒0.7 µm without any coil gap, and coil length of 5‒8 mm for a 2 h reaction time. The growth rate of the CFs that formed the carbon coils was about 7 µm/s and that of the carbon coils was 1 µm/s [7]. Zhang *et al*. synthesized carbon nanocoils (CNCs) in high yield, using iron-coated indium tin oxide as a catalyst and acetylene as a carbon source [101]. They obtained high yield of coils at deposition temperature of 700 °C; and found that the indium, tin, oxygen, and/or their alloys were decisive for the formation of coils. Yang *et al*. prepared carbon microcoils (CMCs) with changing coiling chirality using fine powders of WS_2_ and stainless steel plate as catalyst by CTCVD method [89]. Qin *et al*. prepared amorphous HCNFs with absolutely symmetric structures by polymerization (about 90%) and decomposition (about 10%) of acetylene over copper tartrate as acatalyst [102]. The reaction was carried out at low temperature of about 250 °C and gave good reproducibility and yield. They noticed that there were always two coiled CFs (CCFs) grown on a single copper nanocrystal. Interestingly, these two CCFs were identical in fiber diameter, coil diameter, coil pitch, coil length, and cycle number; however, they had opposite helical senses. CNCs have been synthesized using catalytic pyrolysis of acetylene in the temperature range of 740‒770 °C [8]. Here, two conditions such as sputtered thin films of Au and Au/Ni were used as catalyst and a magnetic field was applied to the synthesis region of CNCs. Coil-in-coil CNCs have been synthesized by means of acetylene decomposition over nickel nanoparticles as catalysts [50]. In this study, CNCs self-assembled in one nanospring and the yield of CNCs was 11 g in each run at the synthesis temperature of 450 °C [50].

#### 2.1.2. Microwave CVD

The functional materials can be prepared at lower temperatures and for shorter times with improved properties using microwave CVD method. The main advantageous feature of this method is that there is no need for impurity element in reaction zone for the synthesis of HCFs [90]. Xie *et al.* adopted a new approach to synthesize the micro CCFs using microwave CVD method [90]. In this study, they used a silicon carbide substrate since SiC absorbs microwaves very efficiently; and they did not use any toxic impurity gas as a growth promoter for HCFs. Their microwave system was composed of a switching power supply, which drives a 3kW magnetron, a circulator, stub-tuner, and a single mode cavity. The micro CCFs obtained by the microwave method was highly coiled and disordered (amorphous).

#### 2.1.3. Hot Filament CVD

HCFs have been synthesized by a hot filament assisted CVD at a substrate temperature of 600 °C using cobalt and copper alloy films as catalyst [73]. In this study, the catalysts were prepared by thermal evaporation coating of a thin layer (20 nm) of cobalt and copper (Co:Cu = 5:1 by weight) on a SiO_2_/Si(110) (thermally oxidized, 1 µm thick SiO_2_) wafer. The metals were simultaneously evaporated with a tantalum boat filament in 2 × 10^−7^ Torr pressure. After coating with metal layers, the substrates were introduced to the CVD system and heated to 600 °C in argon ambient. The heating rate was 100 °C/15 min and annealing of the substrates was carried out for 1 h. When the annealing process was completed, the filament (*W*, diameter = 0.5 mm) was heated to a temperature of 2000 °C, and 5% methane with argon was introduced to the system for 1 h. After completion of the synthesis, the samples were allowed to cool to room temperature while argon flowed through the system.

### 2.2. Arc Discharge

Zhang *et al*. synthesized carbon coils by decomposition of acetylene using arc discharge method under different gas pressures from 160 to 460 Torr over a metal plate [86]. They mentioned the advantageous feature of their method is that there is no need for the complex preparation of the catalyst particles and the introduction of the impurities. Their experimental apparatus is composed of a spectral pure graphite rod as an anode, a metal plate as cathode, acetylene as carbon source, reaction chamber, vacuum pump, circulatory system, DC power supply, *etc*. At a voltage of 35‒40 V and an output current of 96 A, an arc was generated in an acetylene atmosphere and at a pressure of 160‒460 Torr. After three minutes, the club-shaped products of sizes approximately 1 mm, deposited vertically over the surfaces of the plate.

### 2.3. Purification of HCFs

Mostly the as-synthesized HCFs contained metal catalyst particles as impurities, causing problems in property characterization as well as applications of HCFs. Purification of HCFs were usually a tough, lengthy, costly, and destructive process, and introduces defects into the purified sample [91]. However, in some cases, the presence of metal catalysts makes the as-synthesized HCFs advantageous in some fields such as catalyst, catalyst support, electromagnetic wave absorption, *etc*. Mostly acid treatment was applied for the purification of HCFs. Jaybhaye *et al*. removed amorphous carbon and metal particles from the as-synthesized spiral CNFs using acid treatment [45]. For removing impurities, the as-synthesized material was soaked in either conc. HNO_3_ or conc. HCl for 3 h. Then the material was washed with water till neutral pH. Finally, to remove the residual water, the sample was rinsed with acetone and then heated in an oven at 150 °C. Then the purified sample was heated up to 700 °C in hydrogen atmosphere for 2 h to oxidize the amorphous carbon [45]. Therefore, they obtained the purified spiral CNFs. For removing copper nanoparticles (Cu NPs), Li and Xu soaked their CNF samples in concentrated HNO_3_ acid for 1 h, separated by centrifugation, and washed with deionized water until the pH of the filtrate was neutral [67]. Further, the sample was washed with absolute ethanol and dried at 80 °C and purified sample were obtained. In order to avoid the complicated purification process of the as-synthesized material, Qi *et al*. used water-soluble salts NaNO_3_ as catalyst for the synthesis of high-purity linear and helical CNFs [91]. Since NaNO_3_ is water-soluble, it was removed from the as-synthesized materials through a washing process with deionized water, and high-purity HCNFs were obtained easily and undamaged. Ding *et al*. also used water-soluble Na_2_CO_3_ powder as catalyst for the synthesis of nitrogen-doped (N-doped) CNFs (N-CNFs) and N-doped CNCs (N-CNCs) at 450 and 500 °C, respectively [82]. Due to water-soluble property of NaCO_3_, through repeated washing with water and ethanol, there is complete elimination of Na_2_CO_3_ as well as ethanol-soluble organic impurity, and the products were obtained in high purity.

## 3. Effect of Experimental Parameters on the Growth of HCFs

Experimental parameters such as nature of catalyst [73,103], composition of catalyst [104], particle size of catalyst [68], type of carbon source [7], flow rate of gases [55], pressure of gases [86], synthesis temperature [67,85], and active shape (final shape) of the catalysts during synthesis [72,105], are the key parameters, which establish the growth process, structural morphology and microstructure of the as-synthesized HCFs.

### 3.1. Effect of Preparation of the Catalysts for the Growth of HCFs

**Figure 5:**
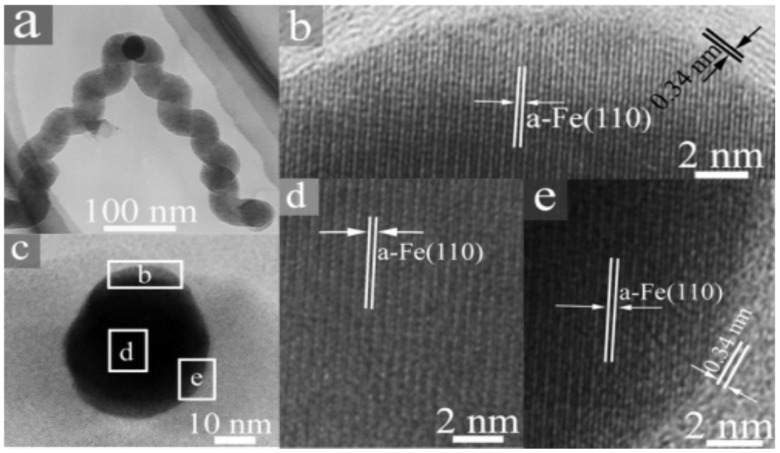
Microstructure of a catalyst nanoparticle located at the node of HCNFs; (**a**) transmission electron microscopy (TEM) image; (**c**) high resolution transmission electron microscopy (HRTEM) image; (**b**, **d**, and **e**) magnified images of areas marked in Figure 5(**c**), respectively ([57], Tang *et al*. 2006).

Several researchers have been prepared special type of catalysts for synthesizing HCFs by adopting several techniques. These techniques include addition of additives or mixture of metal catalysts, use of a support, pretreatment of catalyst in a particular environment, using catalyst precursor, *etc*. These techniques may have considerable effects on carbon deposition, and HCFs with high relevant characteristic can be obtained using special type of catalysts. However, preparation of special type of catalysts is sometimes quite complicated and long process. Qin *et al*. prepared copper tartrate catalyst precursor by the slow addition of 50 mL of 0.1 M copper dichloride aqueous solution to 50 mL of 0.1 M sodium-potassium tartrate aqueous solution under vigorous stirring [61]. After that, nanocopper catalysts were obtained using thermal decomposition of copper tartrate at 250 °C. The nature and grain sizes of these Cu NPs were crystalline and about 50 nm, respectively. They successfully synthesized regularly CCNFs using these nanocoppersas catalysts. In another study of Tang *et al*., Fe nanoparticles have been prepared by the combined sol-gel/reduction method [57]. These nanoparticles were used as catalyst and effective for the growth of HCNFs at 450 °C. In comparison to the common Fe particles, the Fe nanoparticles prepared in such a way were decisive for the growth of HCNFs. In this study, there was no need to modify the Fe nanoparticles by any chiral reagent. The as-synthesized HCNFs were crystalline and symmetric, and (110) plane of the Fe particle was the mirror plane (Figure 5). They also observed that compared to HCNFs obtained over nonmagnetic transition metals, samples containing magnetic Fe nanoparticles show higher magnetization. Recently, Jian *et al*. prepared CCFs from Ni nanoparticles using catalytic pyrolysis of acetylene [62]. In this study, they found that, one of the crucial factors to obtain high-purity CCFs is the controllable synthesis of catalyst nanoparticles. By controlling the reaction temperature and NaOH concentration, they prepared controllable Ni nanoparticles using liquid phase reduction of nickel sulfate with hydrazine hydrate. They used a surfactant polyvinylpyrrolidone (PVP) to prevent agglomeration of Ni nanoparticles. In another study, Jian *et al*. controlled the as-synthesized CFs into linear CNFs; and carbon coils including single-helix CNCs (SH-CNCs), single-helix CMCs (SH-CMCs), and twinning double-helix CMCs (TDH-CMCs) [55]. In this study, these various morphologies of CFs were prepared by only changing the N_2_ gas flow rate and all other experimental parameters were unchanged. Li and Xu prepared Cu NPs generated from the *in situ* decomposition of copper acetylacetonate [67]. These Cu NPs have been used for the selective synthesis of HCNFs, planar CNFs, CNTs, and nitrogen doped (*N*-doped) CNFs by adjusting the reaction temperatures or composition of feedstock gas.

### 3.2. Effect of Various Types of Catalysts on the Growth of HCFs

The properties of a nanostructure catalyst influence the CVD synthesis of CNFs [106]. Here, we discuss the effect of various types of catalysts on the growth of HCFs, which has been used by other researchers.

#### 3.2.1. Effect of Transition Metals

Motojima *et al*. used a Ni single crystal plate (10 × 10 × 0.5 mm^3^) as a catalyst for the growth of HCFs [107]. They examined the effect of respective crystal faces of Ni catalysts on coil yield. They observed that coil yield differed based on the crystal faces, the highest coil yield, in order, by Ni (100) > (111) > (110). Qin *et al*. synthesized amorphous HCNFs at very low temperature of 468 K (195 °C) using copper nanocrystal as a catalyst and acetylene as a gas source at normal pressure [92]. These nanocoils exhibit a symmetric growth mode in the form of twin helices. The majority of the angles between the two twin helices are about 70° or 110°. The IR, XRD, Raman, and C/H molar ratio revealed that HCNFs have a polymer-like structure with a weak trans-polyacetylene feature. These helical fibers were a mixture of solid polymers from acetylene coupling and a small amount of carbon from acetylene decomposition [92]. Nitze *et al*. synthesized HCNFs from CVD process at temperature of 550 °C, using C_60_ supported Pd nanoparticles as catalyst and acetylene as carbon feedstock [93]. Here, C_60_ molecules provided a support for separating Pd into nanoparticles. From the shape of catalyst particles and by adopting a simple model, they concluded that the straight nanofibers curl due to blocked carbon diffusion pathways on the catalyst particles.

#### 3.2.2. Effect of Metal Carbides

Motojima *et al*. analyzed phase of the active catalyst for the growth of HCFs using X-ray diffraction (XRD) and electron diffraction pattern [108]. They found that Ni_3_C single crystal phase was decisive for the growth of HCFs, where thiophene was used as impurity gas. Further, Motojima *et al*. examined catalytic effect of various metals such as Ni, Mo, Zr, Ti, *etc*., on the growth of HCFs during the pyrolysis of acetylene [107]. In this study, the presence of corresponding metal carbides was identified by XRD analysis in the metal grain which was located at the tip of HCFs. Metal, sulfur and carbon were presented in the active catalyst and it was confirmed by electron probe microanalysis (EPMA). However, the corresponding metal sulfide phases were not detected by the XRD analysis. Accordingly, Motojima *et al*. suggested that the metal carbide phase containing solid solution of sulfur and metal, is the actual catalyst for coiling of CFs [107].

#### 3.2.3. Effect of Mixture and Alloys of Metals

Pan *et al*. investigated the effect of combination of Fe and indium tin oxide (ITO) on the growth of CNCs [58]. They found that Fe and ITO are very important catalysts in the growth of CNCs. But neither Fe nor ITO can lead to the synthesis of CNCs separately. They found that Fe was essential for the growth of carbon nanotubules, whereas ITO induces their helical growth. In this study, the yield of CNCs was determined by the elemental ratio of tin and indium in the ITO film. Pan *et al*. further examined that the growing point of carbon coils was the interface of ITO and Fe films where catalyst particles are formed [81]. Okazaki *et al*. developed powder catalysts containing oxides of iron, indium, and tin (Fe-In-Sn-O) for the mass production of CNCs using a coprecipitation technique; and these powder catalysts were found to have the same ability as Fe/ITO thin film catalysts to grow CNCs [104]. They found that Fe was required to grow CNCs and fibers. However, Fe_2_O_3_ by itself could not grow both CNCs and fibers, due to the size of Fe_2_O_3_ particles produced in this study. It was found that Sn was required to grow CNCs, and the composition of Sn should be small to grow CNCs in high yield. On the other hand, indium increased the yield of CNCs by adjusting the crystalline sizes of Fe_2_O_3_ and SnO_2_ [104]. Yu *et al*. prepared Cu-Ni alloy nanoparticles by hydrogen arc plasma method [71]. They synthesized regular CCNFs with asymmetric growth mode by catalytic decomposition of acetylene using these Cu-Ni alloy nanoparticles as a catalyst at 241 °C [71]. Jayatissa and Guo synthesized CNTs using Co catalysts [73]. Furthermore, they obtained carbon helixes when the catalysts were changed to Co + Cu. In this study, the formation of carbon helixes is exclusively governed by the addition of Cu into Co catalysts. Au and K metals were used as bi-catalyst to assist thermal decomposition of C_2_H_2_ for the growth of CNCs [54]. In this investigation, Au and K were deposited on graphite foils substrates and studied the different reaction parameters on the formation of CNCs. Here, no carbon nanostructures (CNSs) were grown without the presence of K. On the other hand, featureless C layers were obtained in the reactions using K and substrates only. In contrast, CNSs in the form of nanoparticles, nanowires and nanocoils were obtained using both Au and K deposited on the substrates. Here, K worked as dehydrogenation catalysts to assist deposition of C from C_2_H_2_; and Au as nucleation seeds for the growth of CNSs.

#### 3.2.4. Effect of Supported Catalyst

The substrate/supporter provide good dispersion of catalysts, higher activity, and selectivity towards the growth of CFs. Chen *et al*. prepared single-helix CNCs with various laces patterns on the surface by catalytic pyrolysis of acetylene using Ni metal catalyst supported on molecular sieve [109]. Molecular sieve contained a small amount of Fe impurity and was originated from a raw material of kaolin clay. They found that a trace amount of Fe impurity contained in the molecular sieve was a key factor for the lace growth. It was considered that Fe impurity may poison or restrain the formation of three crystals faces resulting in the growth of single helix coils, and also activates the anisotropic catalytic activity. Here, they also considered that a very strong inner stress is periodically formed between the boundary of two fibers, and thin carbon films may swell to form laces. In the investigation of Ren *et al*. Cu was used as catalyst over different supporters such as SiO_2_, TiO_2_, Al_2_O_3_, and MgO for the synthesis of HCNFs at relatively low temperature of 237 °C [94]. Mostly HCNFs were obtained using MgO as support compared to other three kinds of supports, and by decreasing the ratio of Cu/MgO, the content of HCNFs increases. The optimal results were obtained at the ratio of 1:10. Their investigation also showed that straight or curved CNFs were obtained using Cu catalyst without any support. Jian *et al*. used two types of substrate for the synthesis of CCFs such as graphite and ceramic plate [62]. In this study, although other reaction conditions were unchanged, they obtained regular CCFs with coil diameter of 3 µm and irregular CCFs with diameter of 1 µm, using graphite and ceramic plate as a substrate, respectively. The distortion of helical fiber occurred randomly, indicating that the interaction between catalyst and ceramic substrate differs from graphite substrate. Park and Kim synthesized carbon coils using acetylene as carbon source and SF_6_ as an incorporated additive gas by CTCVD method [110]. In this study, the supporting substrates were pretreated using various methods before carbon coils deposition reaction. Here, the exclusive formations of the nanosized carbon coils were obtained using thermal etching pretreatment of Ni-SiO_2_ substrate with SF_6_. In addition to this, diamond powders pretreatment of Si substrate gives rise to dominant formation of microsized carbon coils after 10 minutes reaction time.

### 3.3. Effect of Catalyst Particle Size on the Growth of HCFs

Only a few studies provide reasonable explanations to the relationship between the initial catalyst particle size and the diameter of CFs. Since particle size has a direct influence on CF diameter, so CF diameter can be modified by changing particle size. Recently, Wang *et al*. synthesized Cu nanoparticles with narrow size distribution by reduction of CuO films produced by atomic layer deposition (ALD), which are used as catalysts for the catalytic growth of CNSs [68]. By proper adjustment of ALD cycle numbers, the size of produced Cu nanoparticles was well controlled. They mentioned that Cu nanoparticles can be obtained by reduction of CuO films in hydrogen (5% H_2_/N_2_) atmosphere. However, in this synthesis, the CuO nanoparticle films are directly used as catalysts for the growth of CNCs without the hydrogen reduction step since acetylene undergoes slight decomposition at high temperature, leading to the release of reducing hydrogen. Uniform CNCs with nearly 100% purity were obtained using 50‒80 nm Cu nanoparticles, while thin straight fibers and thick straight fibers were produced by applying 5‒35 and 100‒200 nm Cu nanoparticles, respectively [68].

### 3.4. Effect of Shape Changes and Final Shape of Catalyst Particles on the Growth of HCFs

In several studies, the initial shapes of catalyst particles were irregular, and after the growth, the shape of catalyst particles on the tip of HCFs is faceted, specific and regular. It suggests that some changes in the shape of catalyst particles must have take place during the growth process. In addition to this, not only the shape change, but due to the particle agglomeration phenomena and the phenomena of minimum energy, the sizes of catalyst particles also change during the growth process [62]. Kawaguchi *et al.* found that diamond-like or polyhedral-shaped catalyst particles were decisive for the growth of CCFs [97]. These CCFs were formed by the crossing or intertwining of the two primary coils, which were grown in the same coiling direction. The active catalyst particles were mainly composed of a Ni metal with polyhedral-shaped and were always observed on the tip of CCFs. Pan *et al*. found that the diameter, pitch, and shape of a carbon coil are related with the catalyst particle at its tip [81]. In addition to this, the shape of catalyst grain also determines the cross-section of CF [13]. Chen *et al*. observed that the cubic-shaped catalyst grains were decisive for the growth of circular coils and slender-shaped rhombus hexahedron catalyst grains were decisive for the growth of flat coils [13]. The shape changes of catalyst particles during HCFs growth were clearly seen in the study of Qin *et al.* [111]. They synthesized HCNFs with a symmetric growth mode by decomposition of acetylene over copper catalyst. In this study, single copper nanocrystals were decisive for the growth of two symmetrically helical fibers. They explained that upon contacting the initial copper nanocrystals with irregular shapes, acetylene began to decompose to form two straight fibers with the irregular tips. At the same time, the irregular tip of copper nanocrystals began to change to regular shape. After transformation of irregular to a regular faceted shape, the two straight CFs ceased to grow and two regular HCFs with opposite helical senses began to grow. If the regular faceted nanocrystals continue to changes shapes during fiber growth, the two helical fibers possibly changed into opposite helical senses, and thus give helical reversals. The shape changes were caused by the changes in surface energy resulting from the acetylene-adsorption on copper nanocrystals [111]. CMCs have been successfully prepared using catalytic decomposition of acetylene over nickel foam as catalyst at temperature of 973‒1073 K [95]. In this study, thiophene was used as a growth promoter. The average initial grain size of Ni in the nickel foam was 5 µm, whereas the fibers diameter of the as-synthesized CMCs was 400‒500 nm. Here, it was observed that catalyst particles at the tip of CMCs were 400 nm in size and rhombohedral in shape. This reveals that the sizes and shapes of catalyst particles undergo a transformation during the reaction time or CFs growth. AB_5_ type hydrogen storage material such as LaNi_5_ alloy has been used as catalyst precursor for the synthesis of HCNFs [72]. During the reaction course, the oxidative dissociation of LaNi_5_ (sizes ~6 µm) occurred and Ni particles (sizes ~1 µm) were produced. By the interaction with C_2_H_2_ and H_2_, Ni particles were fragmented to Ni nanoparticles (sizes ~150 nm) and by the surface reconstruction phenomena it adopted polygonal shape. At temperature of 650 °C, the growth of HCNFs was achieved through these polygonal shapes Ni nanoparticles. So in this study, the final shapes of the active catalyst particles were polygonal for the growth of HCNFs. However, the starting catalyst particles of LaNi_5_ alloy were irregular and bigger in size.

### 3.5. Effects of Reaction Temperature on the Growth of HCFs

Reaction temperatures strongly affect the anisotropy of catalyst grain, and thus morphologies and dimensions of carbon coils [60].

#### 3.5.1. Morphology of HCFs

Liu and Shen used activated CNTs (ACNTs) supported Ni as catalyst to prepare HCFs, since ACNTs have a larger BET surface area and modified surface to support Ni catalyst [96]. In this study, effect of synthesis temperature on the morphology of final products was investigated within the range of 650‒800 °C. Here, for loading of Ni catalyst, ACNTs were impregnated in Ni(NO_3_)_2_·6H_2_O ethanol solution followed by sonication for 30 min. The mass ratio of Ni to ACNTs was 1:10. Accordingly the Ni containing ACNTs were obtained. They obtained irregular double CMCs, regular double CMCs, double and single CMCs, and CNCs at 800, 750, 700 and 650 °C, respectively, using ACNTs supported by Ni as catalyst. HCNFs have been synthesized using cupric tartrate as catalyst and temperature was below 500 °C by CVD method [38]. Here, effect of reaction temperature on the morphology of reaction products was studied. At low temperature of 280, 380 and 480 °C, planar CFs, HCNFs, and thin HCNFs was the main product, respectively. Raghubanshi *et al*. studied the effect of reaction temperature on the growth of HCNFs [72]. They used LaNi_5_ alloy as catalyst precursor and C_2_H_2_ as carbon source for the growth of HCNFs. They found that polygonal and spherical shapes of Ni nanoparticles were produced and decisive for the growth of HCNFs and planar CNFs at temperature of 650 and 750 °C, respectively.

#### 3.5.2. Coil and Fiber Diameter of HCFs

**Figure 6:**
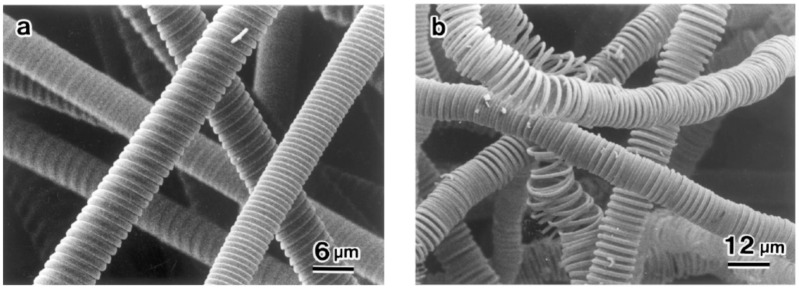
Effect of reaction temperature on the coil diameter: (**a**) regular carbon coils with small coil diameter, reaction temperature 750 °C; and (**b**) carbon coils with larger coil diameter and slightly irregular forms, reaction temperature 820 °C ([60], Chen and Motojima, 1999).

Chen and Motojima observed that the coil and fiber diameter of HCFs were significantly affected by the reaction temperatures [112]. They prepared HCFs by Ni-catalytic pyrolysis of acetylene containing a small amount of thiophene, and investigated the effect of reaction temperature on the coil diameter of HCFs [60]. They found that the diameter of HCFs strongly depends on the reaction temperature. The regular carbon coil with small coil diameter (6‒8 µm) were grown at temperature of 750 °C; and carbon coils with larger coil diameter (12‒14 µm) and slightly irregular forms were grown at temperature of 820 °C (Figure 6). Qin *et al*. observed that the coil diameters of coiled fibers were sensitive to reaction temperature, and could be changed from 100 nm to 10 µm by increasing the reaction temperature [61]. Liu and Shen also observed that diameters of carbon coil decreases while reducing the reaction temperature [96].

#### 3.5.3. Yield of HCFs

Kawaguchi *et al*. investigated the effect of reaction temperature on the yield of HCFs [113]. In this study, HCFs were grown in a relatively narrow temperature range of 600 to 800 °C, while the planar CFs were grown in a wide temperature range of 400 to 1000 °C. The maximum yield (about 30 wt.%) of HCFs was obtained at about 700 °C. No deposition was observed at a temperature below 300 °C. On the other hand, only carbon powder (acetylene black) was obtained above 1000 °C. Ding *et al*. observed that when reaction temperature was higher than 500 °C or lower than 400 °C, the yield of *N*-doped CNCs (*N*-CNCs) was small for their experimental conditions [82]. Above 500 °C, Na_2_CO_3_ (catalyst) decomposes heavily into sodium oxide and CO_2_, this situation is unfavorable for the formation of carbon materials. Also below 400 °C, the decomposition of acetylene becomes adverse [82]. Tang *et al*. used 25 mg, 51 mg, and 163 mg, NiO as catalyst precursor for the synthesis of CNCs at temperature of 415, 425, 450 °C, respectively [50]. They obtained the yield of CNCs was 3.032, 2.41, and 11 g, respectively. So there is a significant increase in the yield of each run upon using a larger quantity of catalyst at higher temperature.

### 3.6. Effect of Type of Carbon Source on the Growth of HCFs

Most researchers have used acetylene (C_2_H_2_) as carbon feedstock for the growth of HCFs [7,38,56,62]. However, many carbon sources other than C_2_H_2_, such as methane (CH_4_), ethane (C_2_H_6_), propane (C_3_H_8_) [114], ethylene (C_2_H_4_), propylene (C_3_H_6_), carbon monoxide (CO), acetone [9], *etc*., have also been used as the carbon source for the growth of HCFs. However, when using hydrocarbons other than acetylene, HCFs were rarely obtained under any reaction conditions [114]. Chen and Motojima prepared HCFs using Ni catalyzed pyrolysis of propane, which was pre-heated at high temperatures, and examined the effects of preheating conditions on the growth of HCFs [114]. In this study, they observed that the growth of HCFs occurred via catalytic pyrolysis of acetylene, which was formed by pre-heating and pre-pyrolyzing of propane. Hence, they concluded that propane can be used as the carbon source for obtaining HCFs if it is pre-heated and pre-pyrolyzed at 1000‒1100 °C to form acetylene. The as-synthesized HCFs were mostly irregular double coils with a large coil pitch of 1‒5 µm and coil diameter of 5‒40 µm [114].

### 3.7. Effect of Reaction Time on the Growth of HCFs

**Figure 7:**
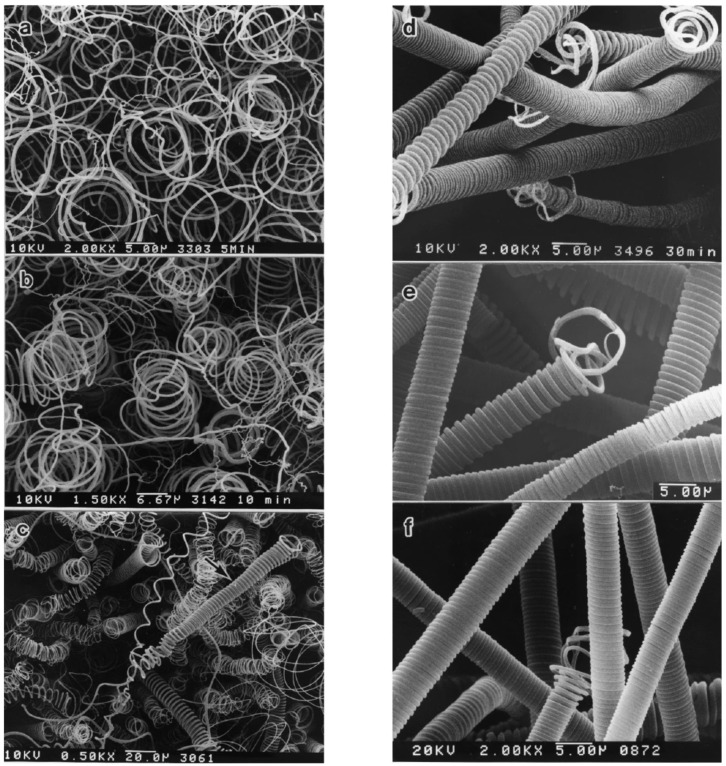
Growth stages of carbon coils. Reaction time: (**a**) 5 min; (**b**) 10 min; (**c**) 15 min; (**d**) 30 min; (**e**) 60 min; and (**f**) 120 min ([112], Chen and Motojima, 1999).

Chen and Motojima investigated the effect of reaction time on the morphology of HCFs [112]. In this study, different morphologies and coiling patterns of HCFs were obtained at different reaction times under standard conditions (Figure 7). It was observed that the clear tips or heads of the carbon coils were formed during 5‒10 min period, but the body part had not been regularly formed. Carbon coils increased in coil length with increasing reaction time. Finally, the regular carbon coils were obtained for 2 h or more reaction time. Chen *et al*. observed that the circular carbon coils are usually formed during the initial growth stage and then gradually changes to the flat coils with increasing reaction time in an electromagnetic field [10].

### 3.8. Effect of Impurity Gas on the Growth of HCFs

Several researchers concluded that the presence of impurity in the reaction atmosphere is indispensable for the growth of HCFs. Kawaguchi *et al*. investigated the effect of impurity gas on the growth of CCFs [113]. In this study, HCFs were rarely obtained using high purity acetylene, but were obtained in high yield using commercially dissolved acetylene. They mentioned that commercially dissolved acetylene contains a small amount of (CH_3_)_2_CO, H_2_S, PH_3_, *etc*. as an impurity. They found that some of these impurities play an important role in the growth of HCFs. In these impurities, only hydrogen sulfide (H_2_S) showed a strong positive effect. For the favorable growth of CCFs, the optimum concentration of H_2_S in acetylene was 0.06‒0.08 vol%. Excessive amounts of impurity may act as a poison on Ni catalyst. Kawaguchi *et al*. analyzed the electron diffraction pattern of the seeds, which were responsible for the growth of CCFs. They found that the seed is a Ni alloy such as a solid solution of Ni and carbon, which also contains small amounts of sulfur impurities. Thus, Kawaguchi *et al*. concluded that a small amount of sulfur impurity acts as an activation agent for a Ni catalyst for the growth of HCFs [113]. Motojima *et al*. examined the effects of various sulfur compounds, such as hydrogen sulfide, thiophene, n-butylmercaptane, dibenzylsulfide, *etc*., as impurities on the growth of CCFs [9]. Among the various sulfur compounds, they found that thiophene was the most effective for the growth of CCFs with a constant coil pitch and high yield. In this study, they also investigated the effect of phosphorus trichloride (PCl_3_) as an impurity on the growth of CCFs [9]. They found that a PCl_3_ impurity significantly accelerates the growth of CCFs. In another study, Chen *et al*. found that no CMCs were formed without sulfur promoter under their preparation conditions [95]. The reason can be explained on the basis of Kim *et al*. [115] consideration, that the presence of sulfur facilitated the catalyst abruption and increased the surface diffusion rate of metal atoms; so by the surface reconstruction phenomena the faceted metal crystallites are formed. It was also suggested that the impurity elements may dissolve into the metal catalyst and cause anisotropic catalytic behavior and thus the formation of CCFs achieved. To avoid the introduction of toxic and unpleasant compound like thiophene, PCl_3_, or H_2_S, *etc*., during the reaction condition and getting the thin film of quasi-aligned CMCs, Ni-sulfide were used as a catalyst by Mukhopadhyay *et al*. [97]. They mentioned that the ball milled Ni-sulfide particles had already the catalytic anisotropy which makes it decisive for the growth of CMCs at 740 °C.

### 3.9. Effect of External Electromagnetic (EM) Field and Bias Voltage on the Growth of HCFs

In-Hwang *et al*. examined the effect of external EM field and bias voltage on the growth of carbon coils [116]. EM field and bias voltage were applied to the reaction tube and substrate, respectively, and significantly affects the growth of carbon coils. Yield of carbon coils with an external EM field and bias voltage were 1.9‒2.0 times higher than that obtained without an EM field and bias voltage [116]. In addition to this, morphology of carbon coils was also changed using external EM field and bias voltage. Kuzuya *et al*. examined the superimposed irradiation effects of EM field (AC, DC) emitted from both the outer and inner sides of the reaction tube on the growth, morphology and some properties of carbon coils [11]. Kuzuya *et al*. found that the coil yield increased by 1.1‒1.2 times with superimposed application of AC or DC, EM field. Also, the density of the carbon coils increased with the application of EM field.

## 4. Synthesis of HCFs-Based Materials

**Figure 8:**
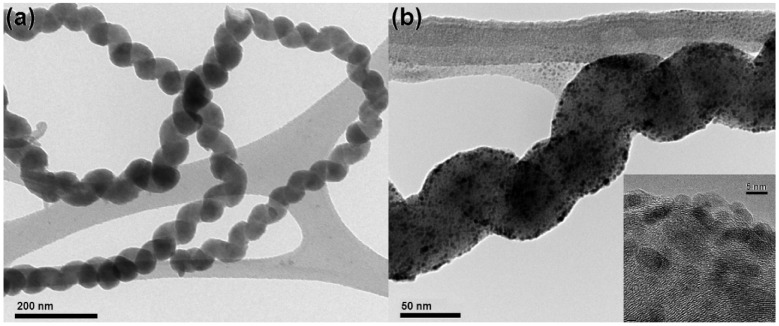
TEM images of (**a**) HCNFs and (**b**) Palladium nanoparticle decorated HCNFs (inset: HRTEM of Pd-HCNFs) ([77], Jia *et al*. 2013).

Electroless deposition has been utilized for the uniform coatings of Ni-Fe-Co-P on CCNFs [78]. In this method, continuous coatings on CCNFs were achieved through proper pretreatments, surfactant and ultrasonic agitation. They found that in comparison with the uncoated CCNFs, the coated ones were fine ferromagnetic materials. In addition to this, the coated CCNFs have shown fine electromagnetic wave-absorbing property in the frequency region of 8‒18 GHz [78]. Ding *et al*. synthesized *N*-doped CNCs (*N*-CNCs) using acetylene as carbon source at 500 °C. In this study, ammonia was used as a source of nitrogen and Na_2_CO_3_ powder as catalyst [82]. Their XRD results showed the decline of graphite signal intensity in CNCs after doping with nitrogen. The reinforcement of rubber has been performed by mixing of HCNFs in natural rubber (NR) [38]. In this study, addition of 100 wt.% HCNFs in NR provides the maximum bound-rubber content (37%). Here, the responsible factor for the amount of bound rubber is the unique coil structure of HCNFs. Jia *et al*. prepared Pd-HCNF nanocomposites by a one-step reduction free method in dimethylformamide (DMF) and applied in hydrogen peroxide and glucose detection [77]. Here, they prepared HCNFs using Pd_2_C_60_ as catalyst in a CVD system followed by functionalization with Pd nanoparticles. TEM image of HCNFs with diameter in the range of 40‒60 nm is shown in Figure 8a. Individual Pd nanoparticles appear on the surface of HCNFs, after co-incubation with Pd_2_DBA_3_ in DMF (Figure 8b). Pd nanoparticles are highly dispersed and well anchored on HCNFs. The sizes of Pd nanoparticles are about 5 nm. The HRTEM image (inset in Figure 8b) shows lattice fringes of both graphitic sheets of HCNFs and Pd nanoparticles.

## 5. Growth Mechanism of HCFs

**Figure 9:**
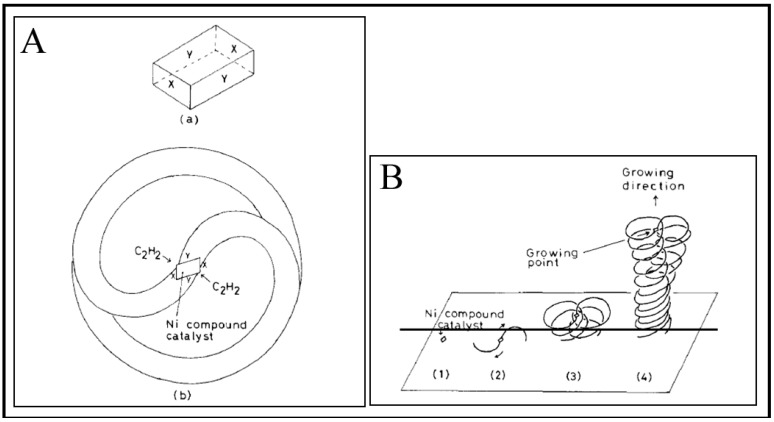
(**A**) Simplified scheme for the growth mechanism of HCF: (**a**) Ni compound seed (single crystal) on tip part of pair-HCF, (**b**) growth mechanism of pair-HCF; and (**B**) growth process of pair-HCF ([113], Kawaguchi *et al*., 1992).

**Figure 10:**
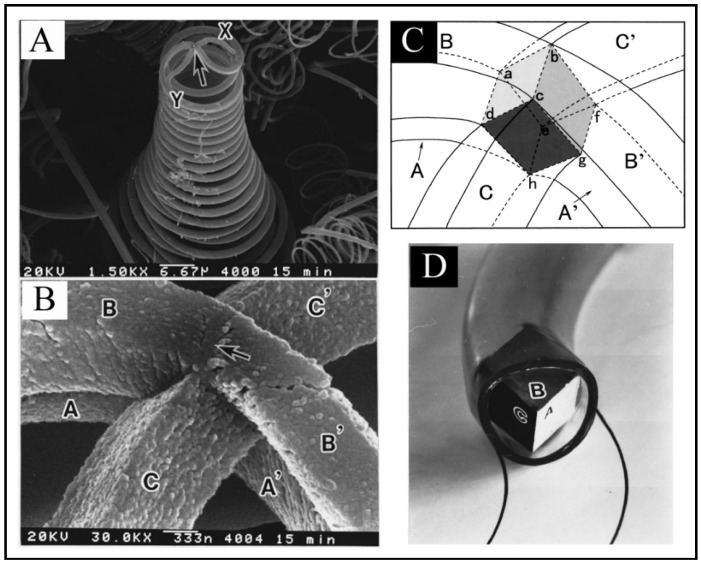
(**A**) carbon coils during the initial growth stage;and (**B**) an enlarged view. Arrow indicates a Ni catalyst grain, *X* and *Y*: paired coils, A, B, C, A’, B’, C’: carbon fibers grown from a Ni catalyst grain (arrow); (**C**) Postulated Ni catalyst grain. a‒h: cubic Ni grain embedded in a node of six fibers; (**D**) 3D growth model of the carbon coils. A‒C: three crystal faces, order of the catalytic activity for the carbon deposition: A > B > C ([7], Motojima and Chen, 1999).

**Figure 11:**
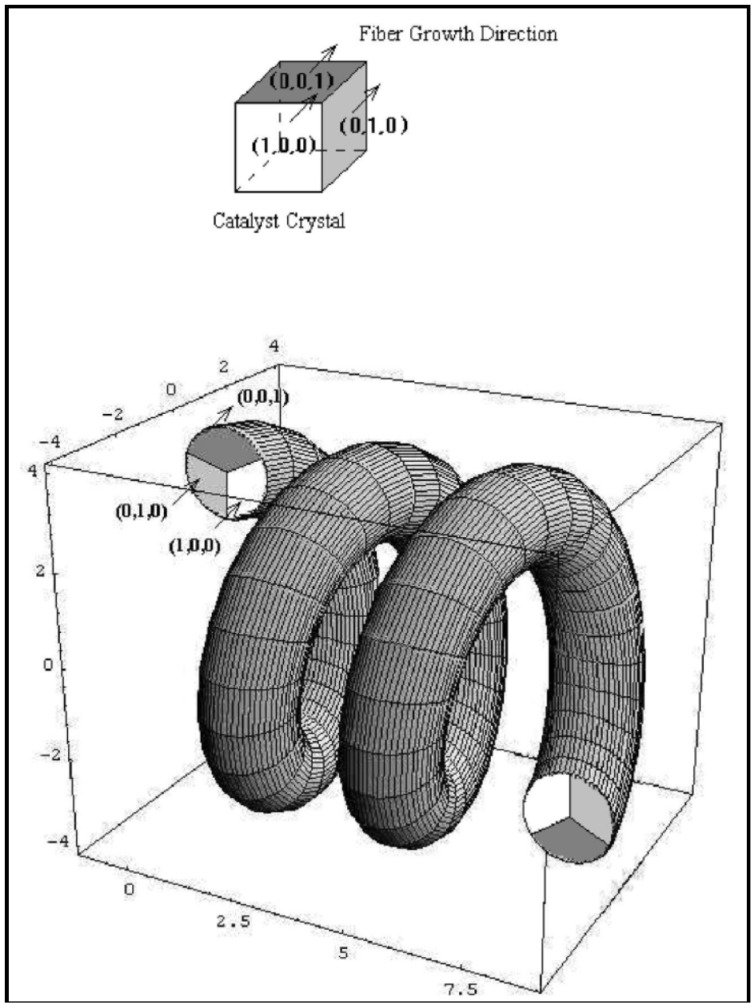
3D model for thegrowth mechanism of coiled carbon nanofibers ([59], Wen and Shen, 2001).

Growth mechanism of HCFs is very interesting. In the growth of HCFs, the rate-determining step is the diffusion of carbon through metal catalyst. In this regard, “anisotropy of carbon deposition” theory, which was proposed in 1992 by Kawaguchi *et al*. [113] for the growth of HCF and further confirmed in 1999 [7], has been the most accepted. In addition to this, many researchers have proposed numerous growth models for HCFs. Some of them are discuss here. Kawaguchi *et al*. proposed a growth mechanism for the pair-HCFs called the “quasi-VLS mechanism”, which involves the surface diffusion of carbon species on Ni compound seed [113]. They observed that Ni compound seed located on the tip of coil fibers is the exclusive growing point and different crystal planes of this Ni compound seed have different catalytic abilities for the growth of HCFs. The opposite planes (*X*-*X*, *Y*-*Y*) in Ni compound seed are equivalent crystallographically, whereas adjacent planes (*X*-*Y*) are not equivalent (Figure 9Aa). According to this theory, initially, a Ni compound seed is formed by the reaction of a Ni particle with acetylene and impurity gas (H_2_S). The decomposition of acetylene were achieved through the catalytic ability of Ni compound seed and then two CFs were start to grow in a direction opposite from each pair of planes (*X* + *Y*) of Ni compound seed (Figure 9Ab). At this stage, due to the different growth rate of each plane, CFs twisted with a curvature corresponding to the diameter of coils. The coil diameter may depend not only on the difference in the growth rate of CFs, but also on the flexibility of CFs. Kawaguchi *et al*. considered that during the growth of CCFs, the surface of Ni compound seed should be in a liquid-like state (quasi-liquid), so that carbon atoms can diffuse rapidly on the surface of the seed and maintain a rapid growth of CCFs. Therefore, Kawaguchi *et al*. proposed that the driving force of the curling of a CF to form HCF is the anisotropic deposition rate of carbon on the respective crystal planes of a catalyst grain. Further, Chen and Motojima explained that the Ni catalyst seed is composed of Ni_3_C single crystals on the surface of which small amounts of sulfur and oxygen are contained [112]. They suggested that the different content of carbon, sulfur and oxygen on the respective crystal faces is the key factor for the anisotropic characteristics of the different crystal planes. Motojima and Chen proposed a three dimensional growth mechanism for CMCs according to the growth patterns and morphologies of carbon coils (Figure 10) [7]. Figure 10A shows a carbon coil obtained during the initial growth stage. The coil diameters increased from about 15 µm at the tip part to 35 µm at the foot. The arrow in Figure 10(A&B) indicates a Ni catalyst grain. This Ni grain is an exclusive growing point for carbon coils. The postulated form of the Ni grain embedded into the node of the six fibers is shown in Figure 10C, in which the Ni grain is shown by a dashed cubic form outline, a–h. In this model, the order of the catalytic activity for the carbon deposition among the three crystal faces is A > B > C (Figure 10D). Basically, a CF is formed from the fine carbon grains deposited from the three crystal faces of A, B, and C, and curl in such a way that the carbon grains deposited from the crystal faces A and B are on the outer surface of the coil, while the grains deposited from the crystal face C are inside surface. The anisotropy of the carbon deposition between the crystal faces of A and C, and/or B and C determines the coil diameters; while the coil pitch by that of A and B (Figure 10D). According to this mechanism, the micro coiling morphology is formed by the rotation of a Ni catalyst grain, from which six fibers grow and coalescence together, followed by the formation of two fibers. These two fibers curl in the same direction around the coil axis and at the same speed of about one cycle per second (Figure 10). Wen and Shen given the 3D growth model for coiled CNFs and coiled CNTs [59]. Their experimental results on CNFs were reasonably in good agreement with the “anisotropy of carbon deposition” theory. In this study, the crystal grain was a Ni-P-Cl co-crystal since they were added to a small amount of PCl_3_ into the acetylene gas [59]. In this model, Wen and Shen assumed that a fiber growing out of three contiguous crystal planes, (1,0,0), (0,1,0), and (0,0,1) with increasing carbon condensation rates, *R*_(0,0,1)_ > *R*_(0,1,0)_ > *R*_(1,0,0)_; according to this rate the final shape of the CF was coiled (Figure 11). The three adjacent crystal planes are labeled in different shades, with the darker one *i.e*., (0,0,1) having a higher fiber growth rate. The resultant fibers grown towards the crystal planes having the lower fiber growth rates *i.e*., (1,0,0), and so coiled shape forms. Amelinckx *et al.* proposed a formation mechanism for a catalytically grown helix-shaped graphite nanotube from a catalyst particle based on a spatial velocity hodograph [18]. Blank and Kulnitskiy proposed a formation mechanism for helically coiled CNFs from a catalyst of octahedral shape [117]. In this mechanism, the sp^3^ bonded carbon atoms, surface tension force, and component of surface tension force played the role for curling the CNFs. Bandaru *et al*. proposed a plausible model for coiling in nanostructure growth motivated by both energy and entropic principles, that for a given volume of material, the helical form occupies the least amount of space [118].

Zhang *et al*. described the formation of carbon coils by giving some external module in addition to the anisotropy of the catalyst grain [86]. In this model, at first, the formation of straight CFs from catalyst particle and generation of van der Waals forces take place. Then, van der Waals force decreases due to change of temperature, and the distortion of fibers arises due to the stress along the fiber axis. Further, the elastic module of the straight fibers mainly controls the coil diameter, coil length, and coil pitch. Finally, angular momentum conservation is the governing factor for the two coils, which derive from the same catalyst grains, and have opposite helix.

## 6. Challenges and Future Prospects

HCFs are still commercially unavailable materials. Synthesis of high reproducibility of HCFs is a tedious task and remains a challenge due to low controllability of experimental parameters. Initially, some researchers were occasionally observed the growth of HCFs during the vapor phase preparation of CFs [51]. The growth of HCFs was generally accidental, in a very low yield and poorly reproducible. Thus, the controllable, reliable, and efficient synthesis of high purity HCFs is of key importance for scientific and industrial applications. In regard to this, a lot of work was carried out for the synthesis of HCFs with high yield and reproducibility [7,17,60]. However, mostly the growth of HCFs required the presence of either large or small amounts of toxic sulfur or phosphorus impurities in the conventional methods. So there is also a challenge to remove these hazardous materials from the synthesis technique of HCFs and provide a green and clean method. Because of the unique helical shape, HCFs have many potential applications such as catalyst, catalyst support, electromagnetic wave absorption material, electronic or electromagnetic nano-devices like in a Micro-Electro Mechanical System (MEMS) [12]. HCFs and their related materials may be applied as the absorbers of cosmic rays, for tunable micro-devices, micro-sensors, the formation of crosslink in nanocomposites, an actuator, activation catalysts for microorganisms, active molds for the preparation of α-helix proteins, hydrogen storage materials, electrode materials, nanoscale springs, *etc*. The electronic states of helically coiled cages of carbon material suggest that it can be a candidate for a superconductor [19]. In this regard, it is believed that HCFs can have a significant application as a structural material.

## 7. Conclusions

HCFs that came in light after the pioneering work of Motojima *et al.* in 1990 are designated as one of the most attractive carbon materials for electromagnetic wave absorbers, field emission, reinforcing the material in composites, supercapacitor electrodes, catalyst support, catalyst, and nanoelectronic applications. In this review article, detail of the synthesis of HCFs and effect of the experimental parameters is clearly addressed. From this review it is evident that catalyzed thermal CVD is the most suitable and widely used method for the synthesis of HCFs and related materials in terms of the product purity, high yield, and reproducibility. Mostly acetylene gas was used as carbon feedstock; and Ni, Fe, and Cu and their mixture were used as catalyst for the preparation of HCFs. The growth process, structural morphology and microstructure of the as-synthesized HCFs are affected by experimental parameters such as synthesis temperature, catalyst, composition of catalyst, particle size of catalyst, type of carbon source, flow rate of gases, atmosphere, and active shape (final shape) of the catalysts during synthesis. The anisotropy of the crystal faces of the catalyst particles are the fundamental reason for the possible coiling morphology in HCFs. Intensive research activities to improve the synthesis methods and conditions, quality, reproducibility, and high yield of HCFs reached to rewarding conclusions because of their unique three-dimensional helical structures and associated properties. Till date, there have been numerous synthesis methods described in the literature to make HCFs, however, absolute control over the helical morphology still remains a challenge.

## Abbreviation

**S. No.**	**Term**	**Abbreviation**
1.	Atomic layer deposition	ALD
2.	Carbon fibers	CFs
3.	Carbon microcoils	CMCs
4.	Carbon microfibers	CMFs
5.	Carbon nanocoils	CNCs
6.	Carbon nanofibers	CNFs
7.	Carbon nanostructures	CNSs
8.	Carbon nanotubes	CNTs
9.	Catalytic thermal chemical vapor deposition	CTCVD
10.	Chemical vapor deposition	CVD
11.	Coiled carbon fibers	CCFs
12.	Copper nanoparticles	Cu NPs
13.	Helical carbon fibers	HCFs
14.	Helical carbon nanofibers	HCNFs
15.	Nitrogen-doped	N-doped
